# Time-Variant Reliability Analysis for Rubber O-Ring Seal Considering Both Material Degradation and Random Load

**DOI:** 10.3390/ma10101211

**Published:** 2017-10-20

**Authors:** Baopeng Liao, Bo Sun, Meichen Yan, Yi Ren, Weifang Zhang, Kun Zhou

**Affiliations:** 1 School of Reliability and Systems Engineering, Beihang University, Beijing 100191, China; lbp2016@buaa.edu.cn (B.L.); sunbo@buaa.edu.cn (B.S.); 18241892584@163.com (M.Y.); 08590@buaa.edu.cn (W.Z.); 2 Southwest Technology and Engineering Research Institute, Chongqing 400039, China; zkdudu@163.com

**Keywords:** rubber O-ring seal, material degradation, random load, time-variant reliability, finite element analysis

## Abstract

Due to the increase in working hours, the reliability of rubber O-ring seals used in hydraulic systems of transfer machines will change. While traditional methods can only analyze one of the material properties or seal properties, the failure of the O-ring is caused by these two factors together. In this paper, two factors are mainly analyzed: the degradation of material properties and load randomization by processing technology. Firstly, the two factors are defined in terms of material failure and seal failure, before the experimental methods of rubber materials are studied. Following this, the time-variant material properties through experiments and load distribution by monitoring the processing can be obtained. Thirdly, compressive stress and contact stress have been calculated, which was combined with the reliability model to acquire the time-variant reliability for the O-ring. Finally, the life prediction and effect of oil pressure were discussed, then compared with the actual situation. The results show a lifetime of 12 months for the O-ring calculated in this paper, and compared with the replacement records from the maintenance workshop, the result is credible.

## 1. Introduction

Seals are widely used in hydraulic systems. The O-ring used in hydraulic systems can prevent the leaking of hydraulic oil and protect the piston from coming into contact with the inner wall of the cylinder block and being scratched [1,2]. The traditional reliability analysis methods only focus on the material or load with a single variation on the O-ring, which does not reflect the influence of both material degradation and random load. Thus, on the basis of existing analytical methods, the reliability analysis of the O-ring can improve the accuracy of reliability evaluation and ensure the safety of the structure with the consideration of both material degradation and random load.

ASME (American Society of Mechanical Engineers) and ASTM (American Society for Testing and Materials) published the standards of seal rings based on material performance and sealing performance, which separately defining the material reliability and seal reliability [3,4]. These standards provided evidence for analyzing the reliability of the O-ring. Besides, many researchers have studied the reliability analytical methods for the O-ring. Lee [5] and Huang [6] tested the rubber material and worked out the mechanical behavior of the rubber material. Shen et al. [7] studied the mechanical behavior of rubber material influenced by long-term aging and cycling. Based on the studies of the mechanical behavior of rubber, the degradation process of rubber and the material reliability is studied. Marco [8] and Henning [9] obtained the degradation process based on the dissipated energy accumulating in the rubber fatigue circles and the field effect. Liu [10] and Okpin [11] studied the degradation rule of rubber material based on the general accelerated degradation test (ADT) model of the Wiener process and the finite element model, which improve the accuracy of material reliability. On the basis of the degradation process and material reliability, Woo [12] proposed a life prediction method for the O-ring with the help of the finite element method. Fang et al. [13] calculated the reliability of rubber components under random loads on the stress-strength interference model. Furthermore, in the study of load on the O-ring, Zuo et al. [14] studied the influence of the working efficiency on the seals of the hydraulic systems. Li et al. [15] analyzed the maximum stress based on the finite element method. In terms of contact mechanics, Wei [16] and Gambino [17] analyzed the mechanical behavior in the contact surface of the seal and calculated the influence of the contact force on the seal performance. Moreover, other researchers have suggested many other analytical methods, such as calculating the reliability of the O-ring according to the distribution characteristics of contact stress under linear loads [18]. The methods mentioned above can simplify the calculation of the reliability of the O-ring. However, the influence of material degradation and random load in the working environment of the O-ring cannot be omitted. Of the existing studies, none of them has taken into consideration both material degradation and random load over time. Thus, studying the dual conditions of material degradation and the load random distribution can further enhance the accuracy of reliability evaluation.

Regarding the complexity and feasibility of reliability analysis under dual conditions at present, Mejri and Cazuguel [19] have verified the feasibility of the time-variant reliability method and finite element analysis. However, they have not worked out the relationship between reliability and time variation. Jiang and Ni [20] proposed the time-variant reliability method of the structure, which is conducted according to the non-probabilistic analytical model of dynamic structural reliability based on Monte Carlo simulation, but this method is too complicated, consuming much time to complete the entire calculation process. Therefore, it is necessary to combine the existing research and conduct appropriate optimization, in order to combine the dual conditions of material degradation and random load. This can be performed with the help of time-variant reliability methods and the discussion of the variations in reliability trends under fluctuating parameters in order to ensure the analysis accuracy of O-ring reliability when the hydraulic system is at work.

In this paper, we combined the analysis of the O-ring in the works cited above and conducted further analysis. In Section 2, the methods for reliability analysis will be described. In the third section, the parameters required in the reliability model will be obtained by experiments and simulations. In Section 4, based on the results in Section 3, the reliability of the O-ring will be calculated when the parameters are in a submissive distribution. Furthermore, an influential analysis is conducted under life prediction, oil pressure and compared with the actual situation to verify the accuracy. In the last section, some conclusions will be raised for the whole paper.

## 2. Methods for Reliability Analysis

### 2.1. Failure Modes and Criterion

**Figure 1:**
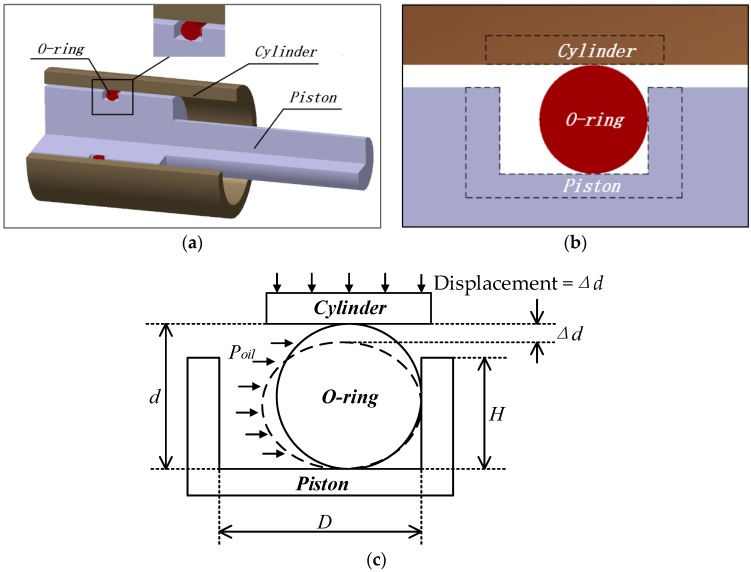
Model of the O-ring: (**a**) solid model; (**b**) part of the area; (**c**) geometric model.

Part of the structure of the hydraulic system containing the O-ring seals is shown in Figure 1a. We extracted a plane from the structure as shown in Figure 1b and established a two-dimensional model during the analysis. This part of the hydraulic system is made of three parts: the O-ring, cylinder and piston. Figure 1c is the geometric model, which includes the structure, geometric size, as well as the shape before and after deformation, within which the O-ring is the main subject of this paper. In one certain type of hydraulic system, some parameters for the model of the O-ring are shown in Table 1.

Before confirming the failure mode of the O-ring, we created three hypotheses in regards to the analysis of the O-ring with the geometric model:

(1). Rubber is the material used in O-rings, which is isotropic and incompressible. The volume of rubber remains the same during its deformation;
(2). As is shown in Figure 1a, the hydraulic system is completely axisymmetric, which means that all the cross profiles of the system bear the same stress. To reduce the computation and increase the accuracy of results, one cross profile (similar to Figure 1b) has been extracted, and the three-dimensional sealing device is changed into a planar question to make the analysis easier;
(3). When constructing the geometric model of the sealing device, we regard the piston and cylinder as rigid bodies, which means when the model is compressed, only the O-ring will deform.

In summary, the O-ring bears the responsibility to perform the function of sealing the hydraulic oil. There are two main types of failure modes. One is the irreversible failure of rubber material when the maximum compressive stress $\sigma_{s}^{max}$ exceeds its limit stress $\sigma_{lim}$. This is due to a stress concentration during the compression of the O-ring. The other is seal failure, which is caused by a change in contract stress. It would happen only when the oil pressure exceeds contact stress [3]. In other words, the seal fails when both $P_{c1}^{max}$ (the maximum contact stress is between the O-ring and cylinder) and $P_{c2}^{max}$ (the maximum contact stress between the O-ring and piston) are lower than oil pressure $P_{oil}$.

Other factors that impact the O-ring, such as fatigue and cyclic load, ultimately lead to material failure and seal failure, and the material failure and seal failure studied in this paper are the most direct failure modes of the O-ring. Besides, as the O-ring is replaced frequently, fatigue and cyclic load are not large enough to have a greater impact on it. Therefore, the influence of other factors, including fatigue and cyclic load, compared with compressive stress and contact stresses, is very small. Therefore, we take compressive stress and contact stress as the main failure criteria for the O-ring in this paper.

### 2.2. Materials

In order to analyze the reliability of the rubber O-ring seal, it is necessary to perform some experiments on the material parameters for the rubber material. However, the data obtained from the experiments cannot be directly applied to the reliability analysis, and this needs to be expressed in the form of a mathematical model. In this section, the mechanical and degradation experiments are performed to determine the performance parameters of the rubber material over time.

Being different from metal material, rubber is a hyper-elastic material. Its parameters are generally defined by the constitutive model. In subsequent simulation calculations, the constitutive model of hyper-elastic materials, such as the Mooney–Rivlin model, the Neo–Hookean model and the Yeoh model, can be directly defined in the simulation. Due to the Mooney–Rivlin model being widely used and its accuracy being recognized by many researchers, the Mooney–Rivlin model is chosen for defining the parameters in this paper [21,22].

The constitutive model is generally expressed in the strain energy density equations for hyper-elastic materials. In our model, the strain energy density equations of the Mooney–Rivlin model of rubber material is:

(1) $$W=C_{10}\left(I_{1}-3\right)+C_{01}\left(I_{2}-3\right)$$

where $I_{1}$ and $I_{2}$ are two strain invariants; while $C_{10}$ and $C_{01}$ are two Mooney constants obtained by stress-strain fitting. The relationship of stress and strain can be obtained through:

(2) $$\frac{\sigma_{1}}{2\left(\lambda_{1}^{2}-\frac{1}{\lambda_{1}}\right)}=C_{10}+\frac{1}{\lambda_{1}}C_{01}$$

where $\sigma_{1}$ and $\lambda_{1}$ are the amount of stress and the percentage of strain, respectively. Both parameters are obtained by mechanical experiments. After the experiments, the stress-strain data are used in Equation (2) to obtain the values for the material parameters of rubber, $C_{10}$ and $C_{01}$.

With an increase in working hours, $C_{10}$ and $C_{01}$ will change with the degradation of rubber material. In this paper, we adjusted the environmental factors of the environmental test chamber to make it consistent with the working environment of the O-ring. A total of m rubber samples are enclosed in the chamber, and the degradation time is zero at this time.

Set $t_{0}=0$ to be the initial time. Combined with the actual situation, we removed k samples at each time point of $t_{1},t_{2},\cdots ,t_{n}$ to perform mechanical experiments. Taking the $t_{1}$ time as an example, k samples were utilized to obtain k group stress-strain curves. According to the stress-strain curve, the limit stress $\sigma_{lim}$ of the k group rubber material can be obtained. Following this, we used the data as inputs into the Mooney–Rivlin model in order to obtain the two Mooney constants $C_{10}$ and $C_{01}$. Compared with the k groups’ stress-strain data, the minimum limit stress $\sigma_{lim}$ is selected according to the maximum safety margin, which reflects the performance parameter of rubber material. Essentially, at $t_{1}$ time, the limit stress is ${\left[\sigma_{lim}\right]}_{t_{1}}$, and the two Mooney constants are ${\left[C_{10},C_{01}\right]}_{t_{1}}$.

Therefore, at each time point $t_{0},t_{1},t_{2},\cdots ,t_{n}$ for the rubber material, there is a set of performance parameters such as the limit stress ${\left[\sigma_{lim}\right]}_{t_{0}},{\left[\sigma_{lim}\right]}_{t_{1}},\cdots ,{\left[\sigma_{lim}\right]}_{t_{n}}$ and the Mooney constants ${\left[C_{10},C_{01}\right]}_{t_{0}},{\left[C_{10},C_{01}\right]}_{t_{1}},\cdots ,{\left[C_{10},C_{01}\right]}_{t_{n}}$. There are n+1 groups of data of limit stress and material parameters.

### 2.3. Reliability Modeling

**Figure 2:**
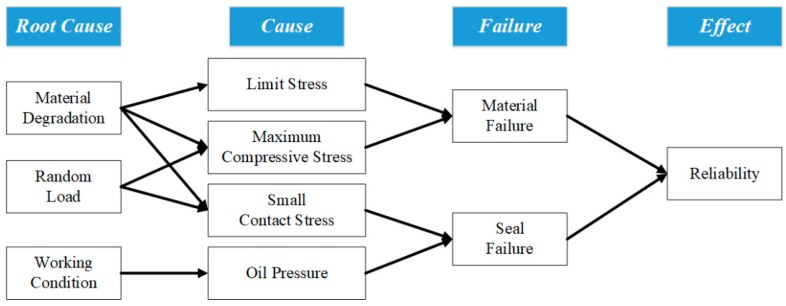
Factors that affect reliability.

With an increase of the working time, the rubber material of the O-ring will degrade, which will result in the variation in the limit stress, maximum compressive stress, etc. However, the random variation of the loads on the O-ring will result in the variation of the relative parameters. Material degradation and random load will finally result in the variation of reliability. Figure 2 shows the variation in parameters caused by material degradation and random load, with the variations in parameters reflected in the changes in reliability in the end.

As shown in Figure 2, we need to solve comprehensively the four intermediate parameters to calculate the reliability of the O-ring: limit stress, maximum compressive stress, small contact stress and oil pressure. Three imported parameters are needed to obtain the previously described four parameters: material parameters of rubber, decrement and oil pressure. This relationship cannot be directly solved through analytical methods, while the simulation can be easily solved. Therefore, simulation and analytical methods can be combined and then express the relationship between the parameters through the response surface method; the reliability of O-ring can be quickly obtained.

In addition to the impact of material degradation on the reliability of the O-ring, the random load is also a factor that cannot be ignored. The analysis of failure modes of the O-ring is described in Section 2.1. The loads on the O-ring are mainly reflected in both the decrement Δd and oil pressure $P_{oil}$.

When the geometric parameters of the O-ring remain the same, the decrement Δd is decided by the processing technology of the cylinder and piston. The distribution rule of Δd can be obtained through gathering the dimension parameters of the components of hydraulic systems. However, the oil pressure $P_{oil}$ is determined by the reacting force on O-rings produced while the hydraulic system is working. This can be obtained by monitoring of the change in oil pressure when the hydraulic system is at work. The distribution of $P_{oil}$ and Δd can be obtained by the analytical method, on the basis of this method, and $P_{oil}$ is assigned the maximum value for safety reasons, assuming that Δd is subject to a normal distribution [23].

Suppose the probability density function of O-rings’ decrement Δd to be f(Δd). Choose the data of ${\left[C_{10},C_{01}\right]}_{t_{0}}$ at the initial time to define the material parameters, and choose $\bar{\Delta d}$ to define the load parameters of the O-ring. The maximum value of oil pressure is $P_{oil}^{max}$. Thus, the input parameters for the calculation of O-rings’ reliability are shown in Table 2.

Unlike $P_{oil}$ and Δd, the compressive stress and contact stress need to be calculated by simulation. After that, the maximum compressive stress $\sigma_{s}^{max}$ and two contact stresses $P_{c1}^{max}$ and $P_{c2}^{max}$ when $\Delta d=\bar{\Delta d}$ can be obtained ($\bar{\Delta d}$ is the mean of Δd). According to the second failure mode in Section 2.1, we defined the small contact stress $P_{c}^{min}=min\left\{P_{c1}^{max},P_{c2}^{max}\right\}$. We compared $\sigma_{s}^{max}$ and ${\left[\sigma_{lim}\right]}_{t_{0}}$ to obtain whether there is failure in the material. Additionally, we compared $P_{c}^{min}$ and $P_{oil}^{max}$ to analyze whether the seal has failed. At this time, the status of the O-ring is obtained when all parameters are in a static state.

After this, we selected k groups of samples within the bounds of Δd. Calculating $\sigma_{s}^{max}$ and $P_{c}^{min}$, and based on the response surface method, the relationship of $\sigma_{s}^{max}$ and $P_{c}^{min}$ changing against Δd can be obtained. According to the relationship, a better mode exists with a higher degree of response surface to display the relationship between $\sigma_{s}^{max}$, $P_{c}^{min}$ and Δd [24,25,26].

Assuming the function between $\sigma_{s}^{max}$ and Δd as:

(3) $$\sigma_{s}^{max}=f_{1}\left(\Delta d\right)$$

the inverse function of Equation (3) would be:

(4) $$\Delta d=f_{1}^{-1}\left(\sigma_{s}^{max}\right)$$

The material reliability of the O-ring would be:

(5) $$\begin{array}{cc} R_{s1} & =P\left(\sigma_{s}^{max}\leq \sigma_{lim}\right)=P\left(f_{1}\left(\Delta d\right)\leq \sigma_{lim}\right)\\ & =P\left(\Delta d\leq f_{1}^{-1}\left(\sigma_{lim}\right)\right)=\int_{0}^{f_{1}^{-1}\left(\sigma_{lim}\right)}f\left(\Delta d\right)d\Delta d \end{array}$$

In a similar manner, considering the function and inverse function between $P_{c}^{min}$ and Δd, the seal reliability would be shown as Equations (6)–(8).

(6) $$P_{c}^{min}=f_{2}\left(\Delta d\right)$$

(7) $$\Delta d=f_{2}^{-1}\left(P_{c}^{min}\right)$$

(8) $$\begin{array}{cc} R_{s2} & =P\left(P_{c}^{min}\geq P_{oil}^{max}\right)=P\left(f_{2}\left(\Delta d\right)\geq P_{oil}^{max}\right)\\ & =P\left(\Delta d\geq f_{2}^{-1}\left(P_{oil}^{max}\right)\right)=\int_{f_{2}^{-1}\left(P_{oil}^{max}\right)}^{\Delta d_{max}}f\left(\Delta d\right)d\Delta d \end{array}$$

where $\Delta d_{max}$ is related to the geometrical parameters of the hydraulic system, including the decrement when there is a minimum distance between the cylinder and piston.

According to Equations (5) and (8), the interval of two groups of Δd can be obtained, which contribute to the adequate degrees of material reliability and seal reliability, as shown in Equations (9) and (10).

(9) $$\Delta d_{Rs1}\in \left[0,f_{1}^{-1}\left(\sigma_{lim}\right)\right]$$

and

(10) $$\Delta d_{Rs2}\in \left[f_{2}^{-1}\left(P_{oil}^{max}\right),\Delta d_{max}\right]$$

where $\Delta d_{Rs1}$ and $\Delta d_{Rs2}$ are the respective values of Δd when the degrees of material reliability and seal reliability are adequate. The union of these two intervals is:

(11) $$\Delta d_{R}=\Delta d_{Rs1}\cap \Delta d_{Rs2}=\left[f_{2}^{-1}\left(P_{oil}^{max}\right),f_{1}^{-1}\left(\sigma_{lim}\right)\right]$$

where $\Delta d_{R}$ is the value of Δd when the degree of system reliability is adequate. Therefore, the system reliability of the O-ring is:

(12) $$R_{s}=P\left\{f_{2}^{-1}\left(P_{oil}^{max}\right)\leq \Delta d\leq f_{1}^{-1}\left(\sigma_{lim}\right)\right\}=\int_{f_{2}^{-1}\left(P_{oil}^{max}\right)}^{f_{1}^{-1}\left(\sigma_{lim}\right)}f\left(\Delta d\right)d\Delta d$$

At this point, the system reliability of the O-ring has been received when $t_{0}=0$, with further repeats of the above calculation in $t_{1},t_{2},\cdots ,t_{n}$. Following this, the time-variant reliability of the O-ring can be obtained. It should be noted that this paper proposes a method to obtain the reliability of the O-ring by combining the analytical and simulation methods. The model input is from the monitoring results or experiment results, which can be calculated by the analytical method. The model calculation is to calculate the stress of the O-ring, which can be obtained by simulation. Finally, the reliability analysis is to process the stress by the analytical method to obtain its time-variant reliability.

## 3. Experiments and Simulations

### 3.1. Experimental Design

The degradation and mechanical experiments of rubber material should be carried out before the simulation. In this section, based on the methods described in Section 2.2, we designed an experimental plan to combining these two experiments, which aimed to acquire the parameters of the Mooney–Rivlin model and the limit stress of rubber material at each time point.

**Figure 3:**
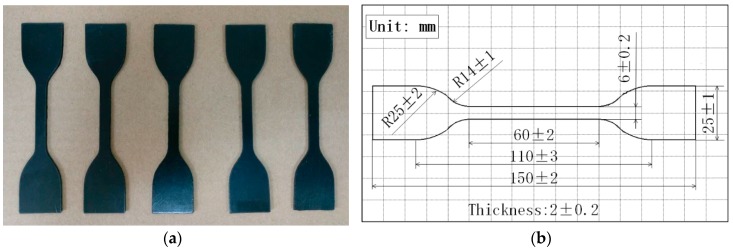
The rubber samples: (**a**) shape of samples; (**b**) dimension of samples.

At the initial time, we placed 35 rubber samples into the container filled with hydraulic oil and soaked them in it, then we placed the container in an environmental test chamber. A group was sampled every three months, with five rubber samples from each group. The temperature of the environmental test chamber is 45 ± 5 °C, and the relative humidity is 50% ± 10%. Furthermore, the shape and dimension of the rubber samples are shown in Figure 3, while the apparatus for experimenting on the rubber samples is a universal testing machine. The stress and strain data in the test are continuously recorded by the testing computer for the universal testing machine.

At the initial time, five samples were removed and underwent a tensile test from the universal testing machine. For safety reasons, we selected the minimum limit stress among the five groups of experimental results as the limit stress of rubber material at this time [27]. Following this, we used the stress and strain data as inputs into the Mooney–Rivlin model by MATLAB (The MathWorks Inc., Natick, MA, USA). Finally, the material parameters include the limit stress ${\left[\sigma_{lim}\right]}_{t_{0}}$ and Mooney constants ${\left[C_{10},C_{01}\right]}_{t_{0}}$, which are obtained at $t_{0}=0$.

The experiments lasted for 18 months and every three months in order to have a different time point. At each time point $t_{1},t_{2},\cdots ,t_{6}$ of rubber material degradation, we removed five samples and repeated the above experiments in order to obtain the material parameters of rubber samples at different degradation times.

### 3.2. Experimental Results

**Figure 4:**
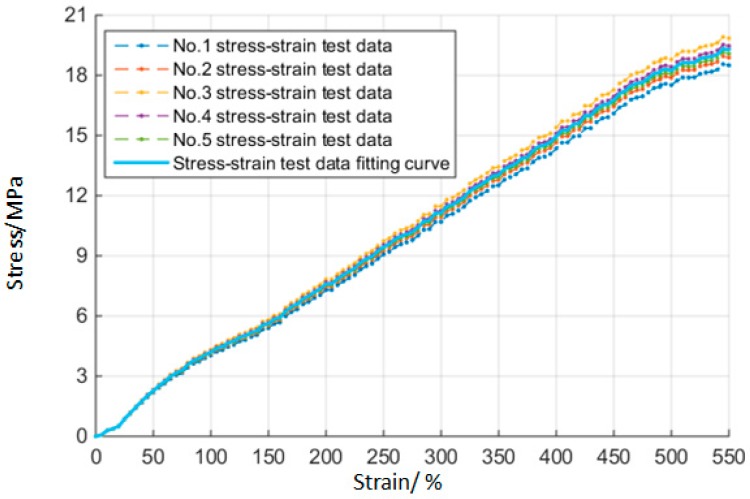
Stress-strain fitting curves for rubber.

Figure 4 shows the fitting curves of stress-strain with rubber materials when the initial time $t_{0}=0$. The minimum limit stress between five samples of rubber material at this time is ${\left[\sigma_{lim}\right]}_{t_{0}}=20.76\;\mathrm{MPa}$. Then taking these data into Mooney–Rivlin model, the Mooney–Rivlin parameters of rubber material at the initial time can be obtained with a value of ${\left[C_{10},C_{01}\right]}_{t_{0}}={\left[10.20e6,\;2.55e6\right]}_{t_{0}}$.

**Figure 5:**
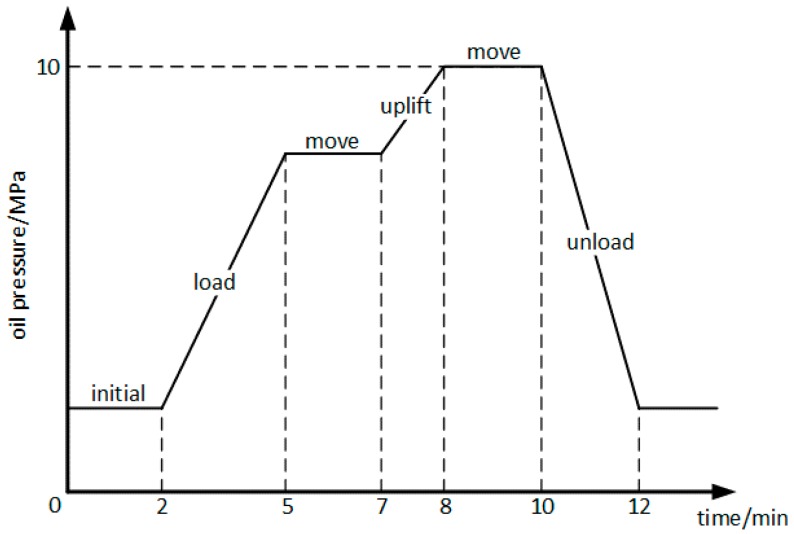
Mission profile of a transfer task.

Following this, by monitoring the variation of oil pressure during operations, the changes in oil pressure in the mission profiles of hydraulic systems can be obtained. Figure 5 shows the changes in the oil pressure $P_{oil}$ during the mission profile. According to the mission profile of a transfer task and the reliability model in Section 2.3, the oil pressure should take its maximum value, which is $P_{oil}^{max}=10\;\mathrm{MPa}$.

### 3.3. Simulations

Although the stress variables (compressive stress and contact stress) can be calculated by analytical methods, the analytical calculation process is particularly complex, so it needs to be deduced and calculated by many formulas, not being suitable for the material degradation and random load in this paper. Therefore, the simulation is selected to calculate the stress variables of the O-ring.

**Figure 6:**
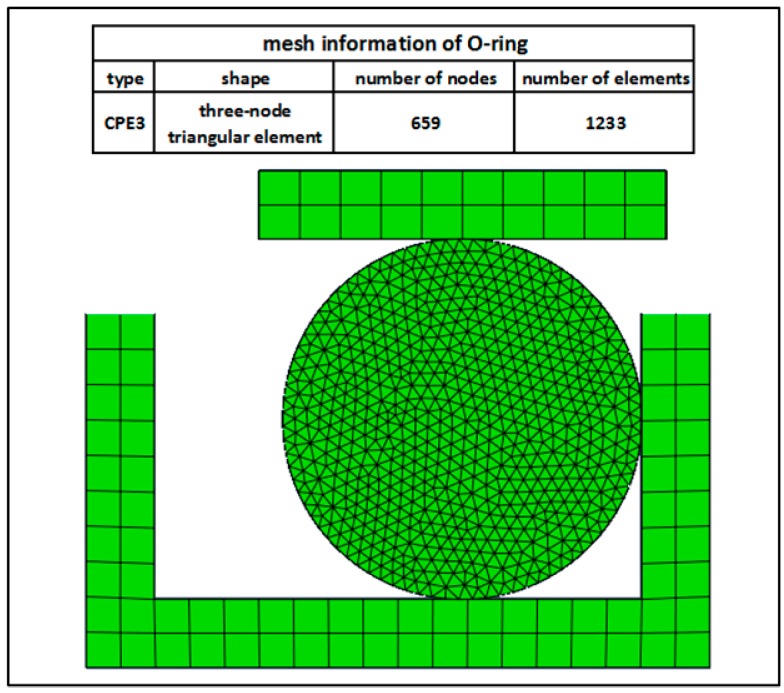
Finite element model of the O-ring.

In hydraulic systems, the piston is fixed, so it has fixed constraints. The cylinder imposes a displacement load with a decrement Δd of 0.3 mm. The O-ring is located in the middle of the cylinder and piston. In ABAQUS (Dassault Simulia Company, Providence, RI, USA), the material properties of the O-ring can be defined directly by the Mooney–Rivlin constitutive model. The two contact surfaces between the cylinder, O-ring and piston are the interacting type of surface-to-surface contact. The mechanical constraint formulation includes the kinematic contact method, while the sliding formulation reflects the finite sliding. Furthermore, the contract properties of two contact surfaces involve the tangential behavior with a friction coefficient of 0.8. Moreover, as the hydraulic systems studied in this paper is not very complicated, consider the computational costs and the use of the refined CPE3 (a 3-node linear plane strain triangle) element, which can fully meet the requirements for the calculation results. Therefore, the meshing during simulation of the O-ring is shown in Figure 6.

**Figure 7:**
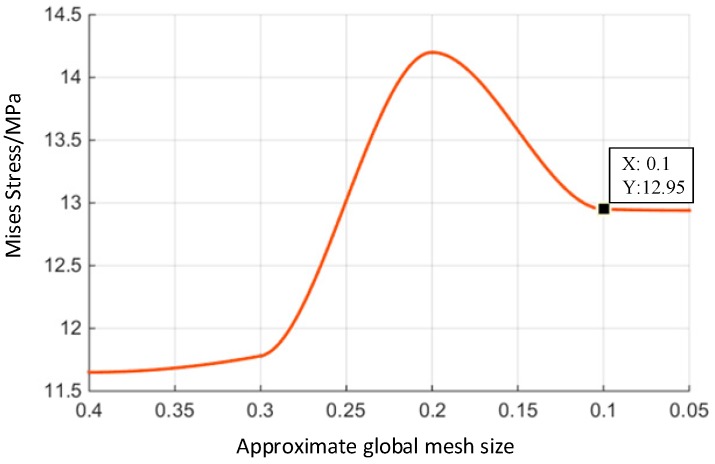
Mesh convergence study.

After pre-processing by ABAQUS, a mesh convergence study is carried out by the finite element model, which uses different mesh sizes. We defined the global size of the mesh to be one, then calculated and compared the results with a step-by-step refinement of the mesh. Figure 7 shows the trend of the Mises stress under different mesh sizes. It can be seen from this figure that the results obtained by different meshes are almost the same when the approximate global mesh size is less than 0.1, so the mesh is considered to be convergent. Thus, it is possible to select the approximate global mesh size of 0.1 as the mesh size during simulation [28].

**Figure 8:**
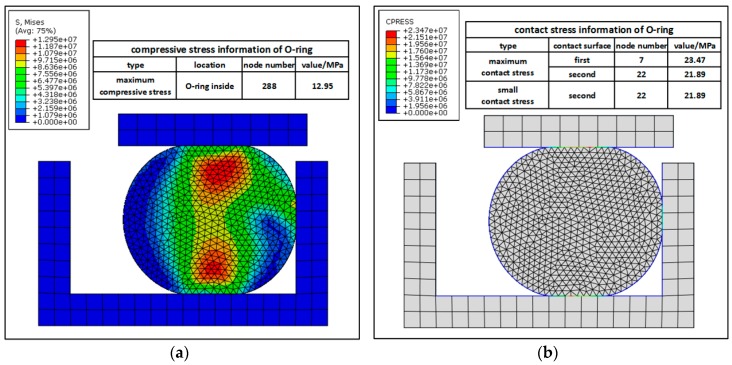
The stress nephogram of the O-ring: (**a**) distribution of compressive stress; (**b**) distribution of contact stress.

Figure 8 is the compressive stress nephogram of the O-ring. Figure 8a shows the maximum compressive stress $\sigma_{s}^{max}=12.95$ MPa. However, the limit stress of rubber material is $\sigma_{lim}=20.76$ MPa. Furthermore, $\sigma_{s}^{max}<\sigma_{lim}$, so the rubber material will not lose efficacy due to stress concentration. Thus, the O-ring is reliable under the present load condition. Figure 8b is the contact stress nephogram of the O-ring. It shows that the maximum contact stress between the O-ring and cylinder ($P_{c1}^{max}$) and that between O-ring and piston ($P_{c2}^{max}$) are respectively 23.47 MPa and 21.89 MPa. As the oil pressure of the sealing device $P_{oil}^{max}=10\;\mathrm{MPa}$, both $P_{c1}^{max}$ and $P_{c2}^{max}$ are larger than $P_{oil}^{max}$. The O-ring performs well in terms of material reliability and seal reliability.

## 4. Reliability Analysis under Multiple Conditions

### 4.1. Material Degradation and Random Load

**Figure 9:**
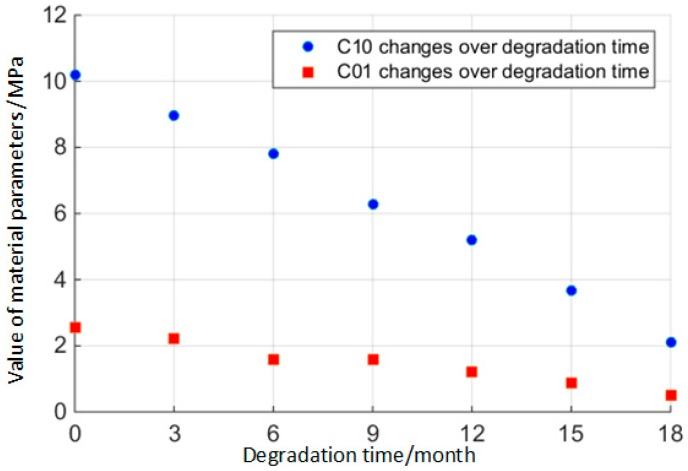
Degradation of rubber material parameters over time.

As the working time of the O-ring increases, rubber material will degrade over time. Using the stress-strain data from the results of the rubber material’s mechanical experiments at every terminal point extracted from the degradation experiments, the rubber material parameters at that time can be obtained with the help of the Mooney–Rivlin model, as shown in Figure 9.

(13) $$\left[C_{10},C_{01}\right]={\left[C_{10},C_{01}\right]}_{t_{i}}\;\;i=1,2,\cdots ,n$$

**Figure 10:**
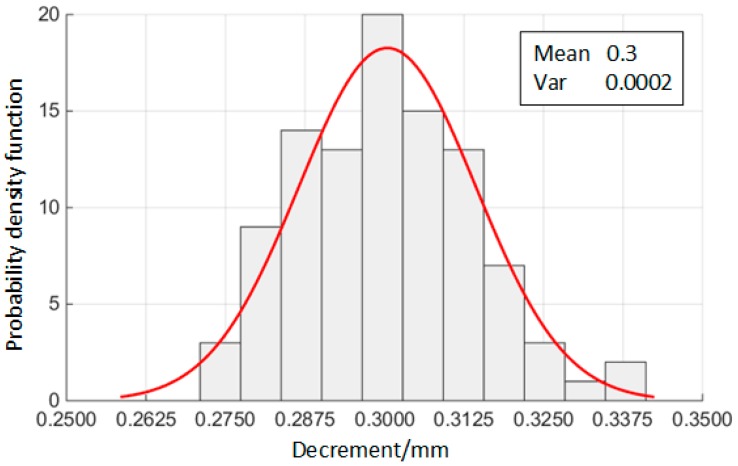
Distribution of decrement.

Following this, in order to decide Δd by processing technology, its distribution rule can be obtained by gathering the size of components. Figure 10 shows the frequency distribution histogram of 100 groups of Δd. Obviously, Δd is of normal distribution.

### 4.2. Result of Reliability Analysis

**Figure 11:**
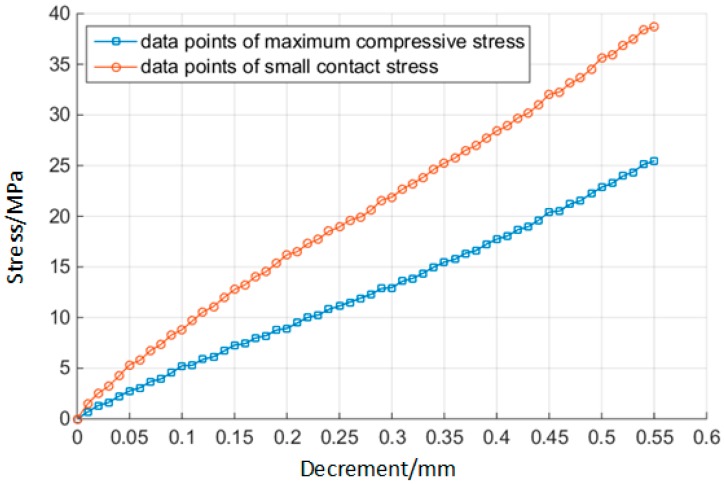
The maximum compressive stress and the small contact stress vary with decrement.

According to the parameters of the geometric model of the O-ring in Table 2, we extracted Δd when Δd∈[0,0.55] every 0.01 seconds. Altogether, there are k=56 groups of Δd samples. The maximum compressive stress $\sigma_{s}^{max}$ and the small contact stress $P_{c}^{min}$ are calculated by ABAQUS. Following this, the two curves of $\sigma_{s}^{max}$ and $P_{c}^{min}$ over the variation of Δd can be obtained, as shown in Figure 11.

In the step of stress variables’ reliability, in the study of this paper, curve-fitting is an analytical method derived from the response surface method, and the accuracy requirements can be guaranteed. Therefore, the curve-fitting was selected to fit the relationship between $\sigma_{s}^{max}$, $P_{c}^{min}$ and Δd. The results are shown in Equations (14) and (15). The degree of fit of the two formulae is 99.8%, which meets the accuracy requirements for estimating $\sigma_{s}^{max}$, and $P_{c}^{min}$ varies with Δd [29].

(14) $$\sigma_{s}^{max}=f_{1}\left(\Delta d\right)=0.2477+44.37\Delta d$$

and

(15) $$P_{c}^{min}=f_{2}\left(\Delta d\right)=1.981+66.77\Delta d$$

Take $\sigma_{s}^{max}$ as an example; Equation (4) can be:

(16) $$\Delta d=f_{1}^{-1}\left(\sigma_{s}^{max}\right)=\frac{\sigma_{s}^{max}-0.2477}{44.37}$$

Thus, the material reliability of the O-ring can be obtained from Equation (5):

(17) $$R_{s1}=\int_{0}^{f_{1}^{-1}\left(\sigma_{lim}\right)}f\left(\Delta d\right)d\Delta d=1$$

Similarly, the seal reliability of the O-ring is:

(18) $$R_{s2}=\int_{f_{2}^{-1}\left(P_{oil}^{max}\right)}^{\Delta d_{max}}f\left(\Delta d\right)d\Delta d=1$$

From Equation (12), the system reliability of the O-ring at initial time can be obtained, that is:

(19) $$R_{s}=\int_{f_{2}^{-1}\left(P_{oil}^{max}\right)}^{f_{1}^{-1}\left(\sigma_{lim}\right)}f\left(\Delta d\right)d\Delta d=1$$

**Figure 12:**
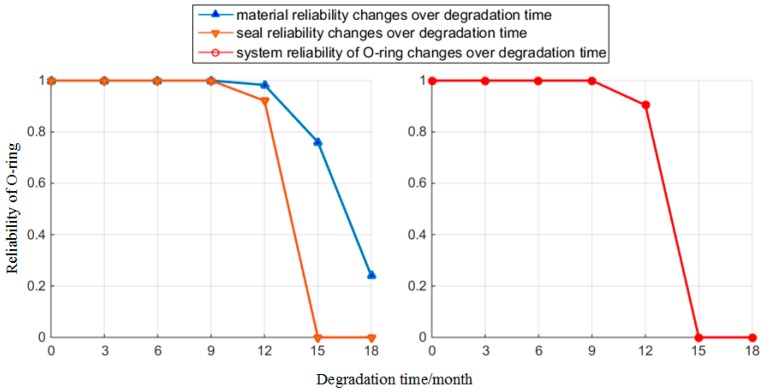
Reliability changes over time.

When the initial time $t_{0}=0$, the reliability of the O-ring is $R_{s}\left(t_{0}\right)=1$. Following this, we used the rubber material parameters at $t_{1},t_{2},\cdots ,t_{6}$ as inputs for the reliability model. Following this calculation, the parameters of the O-ring at each time point can be obtained as shown in Table 3. Furthermore, Figure 12 shows the reliability of the O-ring over the degradation time. Table 3 and Figure 12 can be used to decide that after 12 months of degradation time, the material reliability of the O-ring starts to decrease and drops to 0.2398 at the 18th month. However, the degradation speed of seal reliability is higher than material reliability, and the O-ring totally loses its seal function at the 15th month. O-ring completely failed at this time.

From the calculations at each degradation time point, the reliability of the O-ring remains higher than 0.9 in the previous 12 months. However, in practical work, all types of special states would result in the non-linear fluctuation of parameters, which could lead to serious consequences. Therefore, we analyze its influence on those parameters.

### 4.3. Discussion

#### 4.3.1. Life Prediction and Model Error Analysis

From Table 3 and Figure 12, we can see that the reliability of the O-ring drops dramatically when rubber material degradation reaches a certain time point. However, the production cost of the O-ring is low, and the life cycle cost of replacing its material is even more than replacing the O-ring. On this premise of not replacing the O-ring, according to the results of material degradation and reliability analysis, in order to ensure the safety of the hydraulic systems, we set the replacing period of the O-ring to be 12 months. Moreover, the standard [30] stipulates that sealing element’s overhaul period should not be over 12 months. This paper provides further scientific evidence of quantifying the replacing period. Therefore, we set 12 months as the replacing period of the O-ring to ensure the safety of the hydraulic system in its whole life circle.

Furthermore, the technological limitation would result in variations in the decrement Δd. However, we took the Δd data gathered before and parameters of production equipment into consideration, and the values of Δd are not completely in the normal distribution, but within a certain section [31]. Therefore, regarding the probability density function of Δd in f(Δd), we added a correction factor k and established a new probability density function $f_{M}\left(\Delta d\right)$ for Δd, which is the following:

(20) $$f_{M}\left(\Delta d\right)=\left\{ \begin{array}{cc} kf\left(\Delta d\right) & 0.25\leq \Delta d\leq 0.35\\ 0 & \mathrm{other} \end{array}\right.$$

We used the revised probability density function of Δd to calculate the reliability of the O-ring at $t_{0},t_{1},t_{2},\cdots ,t_{6}$ again. The results are shown in Table 4, which displays smaller changes in reliability compared to Table 3. Thus, processing technology has a low influence on the reliability of the O-ring, and the influence of the processing technology on life prediction is also low. Therefore, the error of the model will not have a greater influence on life prediction, which provides support for the reliability of the method used in this paper to predict the life cycle. Following this, we discussed the influences from other factors on reliability.

#### 4.3.2. Effect of Oil Pressure

**Figure 13:**
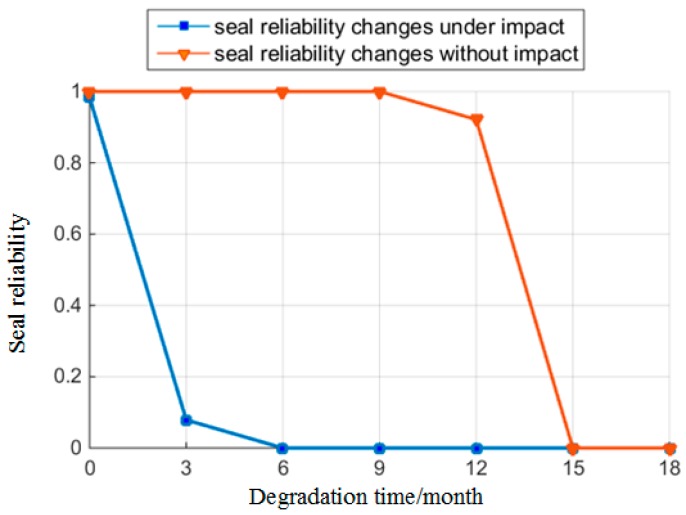
Reliability changes under impact.

In real-life scenarios, loads directly affect oil pressure $P_{oil}$, and incidents outside the mission profile can occur, such as impacts. When the hydraulic system is influenced by the impact load, the instantaneous oil pressure may exceed the maximum oil pressure in the mission profile. We assumed that the maximum oil pressure under impact is twice the maximum value of the original oil pressure $P_{oil}^{max}$, which defines the maximum oil pressure under impact as $P_{oil}^{impact}=20\;\mathrm{MPa}$. The oil pressure mainly affects seal reliability. Therefore, the changing features of reliability are shown in Figure 13 in the situation where the hydraulic systems are under impact load. It can be seen that this impact load is likely to cause leakage in the hydraulic systems within the whole life circle of the O-ring. Therefore, we artificially restricted the probability of impact in the hydraulic systems or used other methods [17], to add several hydraulic systems into parallel connection. These can disperse the impact caused by oil fluctuation and the damage to O-rings when the cost is acceptable. The cost of this method is large, which is applicable to the situations where the risk of leakage is serious.

#### 4.3.3. Comparison with the Actual Situation

According to the reliability model proposed in this paper, the relationship between the time-variant reliability of the O-ring can be calculated, but it also needs to be compared with the actual situation. In order to ensure the applicability of the results between the actual situation and this paper, the working conditions of the actual situation should be the same as experiments and simulation. In the actual situation, the working temperature is maintained at 42 ± 2 °C; the relative humidity is maintained at 48% ± 5%; and the dimensions and load of O-ring are the same as those described in this paper; so there is reason to believe that the working conditions between the actual situation and this paper are the same.

**Figure 14:**
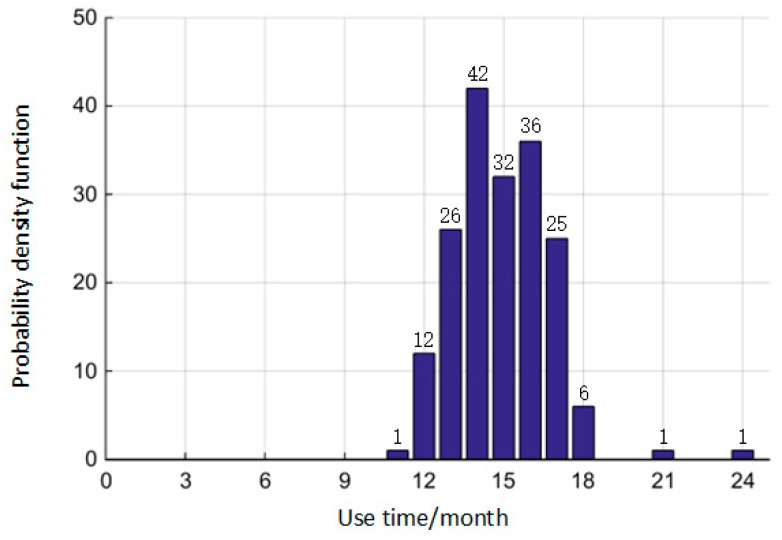
The frequency distribution histogram of use time.

The O-ring works under the mission profile shown in Figure 5, and monitoring the replacement until the O-ring failed, then the statistical data of use time can be obtained. Therefore, we compared the statistical data with the results of this paper to verify the accuracy of the reliability model. Figure 14 shows the use time of the O-ring obtained from the maintenance workshop.

**Figure 15:**
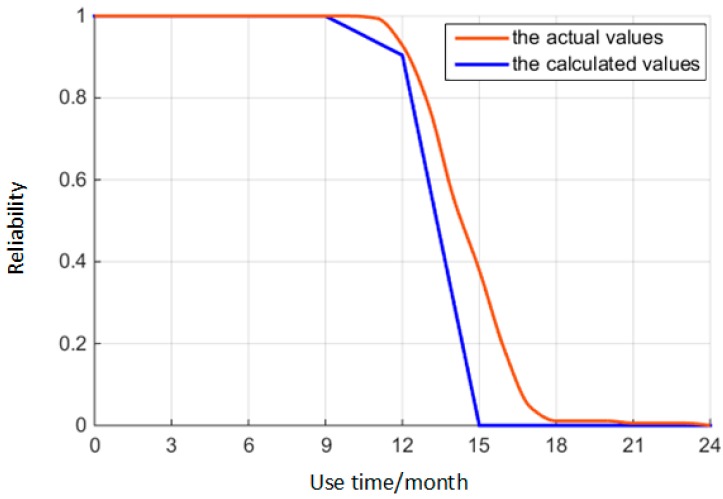
Comparison of calculated and actual values.

By inputting the use time into Equation (21), the reliability of the O-ring can be obtained in an actual situation at various times. These comparison results between the calculated and actual values are shown in Figure 15. The results of this comparative experiment show that the calculated results are similar to the actual results, which verifies that the method of this paper is suitable. Therefore, the reliability model of this paper in predicting the time-variant reliability of the O-ring in the actual situation is accurate.

(21) $$R\left(t\right)=\frac{N-n\left(t\right)}{N}$$

where R(t) is the reliability at time t, N is the number of O-rings monitored and n(t) is the number of O-rings that have been replaced at time t.

## 5. Conclusions

This paper examines the influence of the dual effect of material degradation and random load on the performance of the O-ring. This is conducted by combining the influence of random parameters on reliability from other studies, establishing the finite element model and analyzing the performance of the O-ring. This paper takes multiple factors into consideration, which can overcome the limitation of calculating the degree of reliability with a single variable. Furthermore, the time-variant reliability predicted in this paper considers material reliability and seal reliability at the same time. According to the results of the case studies, the conclusions are as follows:

(1) In view of the time-variant degradation of rubber material parameters, its degradation rule can be obtained by using the experimental method in this paper. According to the experimental results, the performance of rubber material worsens with an increase in working hours.
(2) The maintenance and replacement period of the O-ring predicted in this paper is 12 months, with the number of failures having increased sharply after the 12th month according to the actual situation. There is ample evidence to support why 12 months is used as the replacement cycle for the O-ring. Furthermore, the flaws in the processing technology would lead to varying decrements of the O-ring, despite the variations in decrement caused by the processing technology having little impact on the reliability.
(3) The variation in working load would lead to a variation in oil pressure. Furthermore, the impact load creates considerable damage in the O-ring, which would trigger accidents. We optimized the input of the load and properly distributed the impact load to ensure the safe operation of this hydraulic system.
(4) From the analysis results, the reliability model of the O-ring is obtained and calculated through the case analysis with consideration of both material reliability and seal reliability. In the case study, the reliability of the O-ring is high enough, as confirmed by the actual situation. The method in this paper can accurately and promptly calculate the reliability of the O-ring.

## Figures and Tables

**Table 1:** Parameters of the O-ring.

Parameters	Value
*D* (mm)	3.60
*H* (mm)	2.10
*d* (mm)	2.65

**Table 2:** Input parameters in the calculation.

Input Parameters	Value
$\sigma_{lim}$	${\left[\sigma_{lim}\right]}_{t_{0}}$
$\left[C_{10},C_{01}\right]$	${\left[C_{10},C_{01}\right]}_{t_{0}}$
Δd	$\bar{\Delta d}$
$P_{oil}$	$P_{oil}^{max}$

**Table 3:** Parameters at each time point of the O-ring.

Time/Month	$\sigma_{lim}$/MPa	$\Delta d_{L}$/mm	$\Delta d_{U}$/mm	$R_{s1}$	$R_{s2}$	$R_{s}$
0	20.79	0.12	0.46	1	1	1
3	16.55	0.15	0.42	1	1	1
6	12.86	0.18	0.39	1	1	1
9	9.66	0.21	0.36	1	1	1
12	7.61	0.28	0.33	0.9831	0.9214	0.9044
15	5.06	0.42	0.31	0.7603	0	0
18	2.83	out of range	0.29	0.2398	0	0

**Table 4:** Parameters at each time point of the O-ring.

Time/Month	$R_{s1}$	Deviation	$R_{s2}$	Deviation	$R_{s}$	Deviation
0	1	0.00%	1	0.00%	1	0.00%
3	1	0.00%	1	0.00%	1	0.00%
6	1	0.00%	1	0.00%	1	0.00%
9	1	0.00%	1	0.00%	1	0.00%
12	0.9835	0.04%	0.9217	0.03%	0.9048	0.04%
15	0.7606	0.04%	0	0.00%	0	0.00%
18	0.2399	0.04%	0	0.00%	0	0.00%

## List of Symbols/Nomenclature

**Symbols**	**Nomenclature**	**Definition**	**Unit**
$\sigma_{s}^{max}$	The maximum compressive stress	The maximum value of the stress applied to the rubber during compression.	MPa
$P_{c1}^{max}$ and $P_{c2}^{max}$	The maximum contact stress	The maximum value of the stress generated by the contact surface when contacting each other.	MPa
$P_{c}^{min}$	The small contact stress	The minimum value between $P_{c1}^{max}$ and $P_{c2}^{max}$.	MPa
$\sigma_{lim}$	Limit stress	The stress value of the rubber in the compression process when the normal working capacity is lost.	MPa
$P_{oil}$	Oil pressure	The pressure of the hydraulic oil on O-ring during hydraulic system work.	MPa
$P_{oil}^{max}$	The maximum oil pressure	The maximum value of oil pressure.	MPa
Δd	Decrement of O-ring	Decrement of O-ring during compression.	mm
$\bar{\Delta d}$	The mean of Δd.	The mean of Δd.	mm
$R_{s1}$	Material reliability	The probability that rubber material does not fail.	/
$R_{s2}$	Seal reliability	The probability of no leakage.	/
$R_{s}$	System reliability	The probability of O-ring working properly.	/
$\Delta d_{Rs1}$/$\Delta d_{U}$	The upper limit of decrement	Decrement which satisfies with $R_{s1}$.	mm
$\Delta d_{Rs2}$/$\Delta d_{L}$	The lower limit of decrement	Decrement which satisfies with $R_{s2}$.	mm
$\Delta d_{Rs}$	The limit of decrement	Decrement which satisfies with both $R_{s1}$ and $R_{s2}$.	mm
/	Contract properties	The properties of the two contact surface in ABAQUS.	/
/	Nephogram	A photograph of a cloud.	/
/	Deviation	The degree of deviation from the initial value.	%
